# Protecting the Environment for Self-interested Reasons: Altruism Is Not the Only Pathway to Sustainability

**DOI:** 10.3389/fpsyg.2017.01065

**Published:** 2017-06-28

**Authors:** Stefano De Dominicis, P. Wesley Schultz, Marino Bonaiuto

**Affiliations:** ^1^Department of Business and Management, LUISS Guido Carli UniversityRome, Italy; ^2^Department of Social and Developmental Psychology, Sapienza Università di RomaRome, Italy; ^3^CIRPA – Interuniversity Research Centre for Environmental Psychology, Sapienza Università di RomaRome, Italy; ^4^Department of Psychology, California State University San Marcos, San MarcosCA, United States

**Keywords:** pro-environmental behavior, environmental concerns, message frames, self-interest, altruism, values, sustainability

## Abstract

Concerns for environmental issues are important drivers of sustainable and pro-environmental behaviors, and can be differentiated between those with a self-enhancing (egoistic) vs. self-transcendent (biospheric) psychological foundation. Yet to date, the dominant approach for promoting pro-environmental behavior has focused on highlighting the benefits to others or nature, rather than appealing to self-interest. Building on the Inclusion Model for Environmental Concern, we argue that egoistic and biospheric environmental concerns, respectively, conceptualized as self-interest and altruism, are hierarchically structured, such that altruism is inclusive of self-interest. Three studies show that self-interested individuals will behave more pro-environmentally when the behavior results in a personal benefit (but not when there is exclusively an environmental benefit), while altruistic individuals will engage in pro-environmental behaviors when there are environmental benefits, and critically, *also* when there are personal benefits. The reported findings have implications for programs and policies designed to promote pro-environmental behavior, and for social science research aimed at understanding human responses to a changing environment.

## Introduction

Addressing environmental issues will require that people do things differently (IPCC, 2014). While there has been some success in developing conservation programs that encourage pro-environmental behavior, there remains considerable debate about the most effective strategies. On the one hand, we can make appeals to forgo self-interest, and encourage individuals to engage in behaviors with a more collective benefit (Evans et al., 2013; Schultz, 2001)—both for future generations and for the environment. This is one of the most commonly used strategies in environmental messaging, and in fact, “conservation” is often widely associated with abstaining from a desired action. An alternative approach is to embrace self-interest (Griskevicius et al., 2010), and to develop programs and messages that appeal to the personal benefits of environmental protection, such as saving money or garnering the social approval of others. This approach has been criticized (Bolderdijk et al., 2013), but offers considerable promise in promoting widespread change.

Many environmental issues, such as climate change, can be viewed as a social dilemma (Ostrom, 2010a,b), wherein the interests of the individual are at odds with the collective interests of the group. In a social dilemma—as in related situations such as prisoner’s dilemmas, social traps, and commons dilemmas—a group of individuals with each pursuing his or her self-interest results in worse outcomes for the collective (Dawes and Messick, 2000). For example, driving a personal automobile to work rather than riding a bike may produce individual benefits, such as a faster and more comfortable ride; but the self-interested choice has harmful consequences to the group, such as increased air pollution, greater use of natural resources, and traffic congestion. Solving social dilemmas, especially those related to environmental issues, has been a long-standing matter of debate in the research literature (Messick and Brewer, 1983). Among different variables influencing the occurrence of collaborative vs. non-collaborative behavior in social dilemmas (Dawes and Messick, 2000), research has shown that a person’s predominant motive (self-interest vs. altruism) can have an impact on possible dilemma solutions (Stern et al., 1995; Van Lange et al., 1998; Van Lange, 1999). Within such a framework, it is plausible that according to the specificity of the context, both self-interest and/or altruism can be invoked in the service of the environment (Dietz, 2005). While appeals to altruism have been found to be an effective trigger for promoting pro-environmental behaviors (Bolderdijk et al., 2013; Dietz, 2015), recent developments have highlighted the potential role of self-interest in enhancing pro-environmental action (Griskevicius et al., 2010).

Here, we investigate the effectiveness of different value-based frames to motivate pro-environmental behaviors. We test whether messages oriented toward the enhancement *vs.* the transcendence of the self can produce environmental action in individuals mainly motivated either by self-interest or altruism. In two laboratory and one field experiment, we hypothesize that a self-transcendent message frame is more effective at promoting pro-environmental behavior among altruistically motivated individuals than for self-interest-motivated individuals. However, a self-enhancing message frame is effective in motivating both self-interested and altruistic individuals toward environmental actions. We integrate this main hypothesis into a broader inclusion model for pro-environmental behavior, which explains discrepancies in the literature and can help inform efforts to promote widespread collective environmental action.

The current work draws on the IMEC-Inclusion Model of Environmental Concern (Schultz, 2002; Nolan and Schultz, 2015) and tests the impact of self-enhancing versus self-transcendent appeals to engage in pro-environmental behavior. Following recent research, we differentiate between two types of environmental concerns (Stern and Dietz, 1994; Schultz, 2001; Schultz et al., 2005)—either egoistic concerns (more oriented toward self-interest) or social/biospheric concerns (more oriented toward altruism). According to IMEC, we propose that individuals with egoistically based environmental concerns (henceforth, self-interest) are likely to engage in pro-environmental behavior when presented with a self-enhancing message (but not when presented with a self-transcendent message). However, for individuals with biospherically based environmental concerns (which further expands altruism to include biosphere and all the living things together with other humans—henceforth, altruism), both the self-enhancing and self-transcendent messages increased pro-environmental behavior. In fact, we argue that self-interested concerns are included within the broader and more transcendent altruistic concerns. As a result, making self-enhancing motivators salient is likely to increase pro-environmental behaviors among a broader audience, whereas self-transcendent messages will tend to be motivational only for the subset of the audience with altruistic environmental concerns. This does not mean that policy makers should promote self-interest and eschew altruism, but rather that both frames could be effectively used to promote pro-environmental behaviors according to different yet interrelated social psychological basis. The proposed model could therefore enlarge the understanding of the motivational basis for pro-environmental behavior.

Previous research has argued that values, defined as important life principles and goals that drive a person’s actions, can be classified along the two dimensions of self (from self-transcendence to self-enhancement) and change (from openness to change to conservatism; Schwartz, 1992). While self-transcendence comprises goals that are outside the individual, such as the welfare of other persons or the natural world, self-enhancement comprises goals that promote one’s own interest. Along the second dimension, openness emphasizes a desire for new ideas and new experiences, while conservatism focuses on social stability and tradition. Values in turn provide the basis for environmental attitudes, namely environmental concerns, that are attitudes toward the environment and its related outcomes, differentially oriented toward one’s own self, other people, or nature and all living things (Schultz and Zelezny, 1999; Schultz, 2000, 2001). Therefore, attitudes toward the environment can be differentiated as egoistic, social, or biospheric environmental concerns (Stern and Dietz, 1994; Schultz and Zelezny, 1999; Schultz, 2001; Schultz et al., 2005). Although some research points to environmental beliefs as a form of one’s general value orientation (De Groot and Steg, 2007, 2008), Stern and Dietz (1994) argue that attitudes about environmental issues are based on a person’s more general set of values.

Research has clearly established that self-transcendence and self-enhancement values are predictive of environmental concerns and pro-environmental behavior (Schultz, 2001; Dietz et al., 2005). Self-enhancement reflects a general orientation toward self-interest, defining other people or other living things outside the boundary of self; while self-transcendence reflects a more altruistic orientation, including other people and other living things within the self (Dietz et al., 2005; Schultz et al., 2005). Individuals with strong self-transcendent values tend to have a more altruistic orientation, and are more willing to behave pro-environmentally; while individuals high on self-enhancement are more self-interested and are less likely to engage in pro-environmental behaviors (Schultz, 2000, 2001).

From the evidence cited above, altruism seems the key to pro-environmental behavior. So given this foundation, promoting pro-environmental behavior will require changing or activating self-transcendent values in people, and thereby their more altruistic-based environmental concerns (Bolderdijk et al., 2013). Unfortunately, with regard to changing values, a large body of research has shown that values are relatively stable and difficult to change (Schwartz and Bilsky, 1987, 1990). But perhaps there is a pathway to pro-environmental behavior through the self-enhancing value orientation. Recent findings point to pro-environmental individual choices on the basis of self-enhancing reasons: green behaviors can in fact serve what are usually considered to be self-interested goals, for example to gain reputation or status (Griskevicius et al., 2010), as well as to save money or to improve personal health (Gifford, 2011; Gifford and Nilsson, 2014). In fact, from an evolutionary perspective, behaviors could be biased either toward self-enhancement rather than toward self-transcendence (e.g., behaviors that serve to take advantage on competitors; van Vugt et al., 2014), or vice-versa—for example, when natural selection within groups favored genes that promote pro-social motives (Boyd and Richerson, 2009). Moreover, from an applied-intervention perspective, the effectiveness of pro-environmental communication campaigns based either on self-enhancing or self-transcendent values has not been well established (Thøgersen and Crompton, 2009). In sum, both self-interest and altruism may provide pathways to pro-environmental behavior (Dietz, 2005, 2015).

To our knowledge, at least in the pro-social and pro-environmental domain, only a few studies have examined the relationship between social-psychological variables such as values and attitudes, and message framing. For example, research has shown that self-transcendent messages appeal more to individuals endorsing biospheric values, while self-enhancing messages work best with individuals holding egoistic values (Hansla, 2011; Nilsson et al., 2016). Furthermore, there is evidence to suggest that this effect could be moderated by argument strength, such that when a message is framed in a value-congruent manner, it can result in greater engagement of the receiver (von Borgstede et al., 2014) and it can consequently exert greater persuasive impact (Petty and Cacioppo, 1984, 1986; Johnson and Eagly, 1989). However, additional research is still needed to understand the influence of different message frames on pro-environmental behavior separately for individuals with different types of environmental concerns. With this respect, we reject the position that self-interest and altruism (as well as egoistic and biospheric concerns) are mutually exclusive (Schultz et al., 2005; von Borgstede et al., 2014), and instead we build on an “inclusion” theoretical explanation of the relationship between different environmental concerns.

Specifically, deriving from early formulations of altruism and self-interest (Stern et al., 1993), we build on the Inclusion Model of Environmental Concerns (Schultz, 2002; Schultz et al., 2005; Nolan and Schultz, 2015), suggesting that environmental concerns are organized in a systemic and hierarchical structure (Lewin, 1951; Bronfenbrenner, 1977): egoistic concerns are included within social concerns, which are themselves included within biospheric concerns. This means that some individuals could be mainly self-interested, while individuals who are altruistic are also self-interested; thus altruism might motivate some individuals, while self-interest might motivate many. In other words, to be concerned for the biosphere and for all the living things (altruism) does not happen in the absence of self-interest.

## The Research: Promoting Pro-Environmental Behaviors

To explore this research question, we designed three experiments that targeted different reasons for conserving energy and using public transportation (Experiments 1 and 2) and for participating in a beach clean up event (Experiment 3). According to the Inclusion Model, we expected that when a behavior has an environmental benefit but not a self-interest benefit, this would increase pro-environmental behaviors of altruistically motivated individuals compared to those with a more self-interested motivation, because altruism is not included within self-interest. However, when a behavior has a personal gain, no difference would emerge in pro-environmental behaviors among altruistic or self-interested individuals, because self-interest is included within altruism. In other words, if self-interest is ultimately included within altruism, self-interested individuals will behave green when a self-enhancing reason (but not a self-transcendent one) matches their self-interest, while altruistic individuals will behave green for either a self-enhancing or self-transcendent reason. The experiments’ protocols described below were used to test hypotheses derived from these general assumptions, after they were approved by the Institutional Review Board for the Protection of Human Subjects (IRB) of the California State University San Marcos (CSUSM).

### Experiment 1

#### Aims and Hypothesis

The specific aim of Experiment 1 was to test the hierarchical structure of environmental concerns. In Experiment 1, we used a 2 (within-subjects variable: situational self-enhancing vs. self-transcendent motive) × 2 (between-subjects variable: dispositional self-interest vs. altruism) mixed model experimental design. We test here the effect of situational motive and environmental dispositional concern on the intention to enact pro-environmentally (i.e., save energy and use public transit). Based on our theoretical model, we hypothesized that an individual would be more willing to behave green when a self-enhancing contextual frame matched the person’s dispositional self-interested concern, while both a self-enhancing and self-transcendent situational frames would increase green behavior among individuals with an altruistic dispositional concern (H1).

#### Methods and Materials

##### Participants

A total sample of 124 (female: 73.4%; average age: 22.8 years; ethnicity: 54% White, 25.8% Latino, 3.2% Black, 6.5% Asian, 8.9% Other) undergraduates from California State University San Marcos (CSUSM) were recruited during the spring semester 2014 (January) through the university’s Human Participant Pool. Each signed a consent form and participated in four experimental sessions. Participants received one course credit in exchange of their participation.

##### Experimental procedures

Participants were first asked to complete two unrelated surveys. The first survey measured basic socio-demographic variables and some social-psychological variables, among which were measures of environmental concerns (Schultz et al., 2005). Altruism and self-interest were operationalized in each study by calculating a relative score (difference between “biospheric concern” and “egoistic concern” scores) and then using a median split to classify participants into those with higher relative egoistic scores or relative biospheric scores. In the second survey, in order to test for the within-subjects effect, two scenarios were presented to each participant where we manipulated the situational value frames. For each of the two value frames, two different pro-environmental behaviors (conserve energy and use public transit) were also manipulated and presented randomly. The resulting four sets of combinations (see Supporting Information: Text S1) were randomly assigned to participants, with all the manipulation checks showing results in the hypothesized direction. Specifically, participants reported the self-enhancing scenario to be more beneficial for themselves rather than for the environment, and vice-versa for the self-transcendent one, perceived to be more beneficial for the environment rather than for themselves (all four related *p* < 0.001). By this procedure, two different situations, framed according to each of the two manipulated value frames (self-transcendent vs. self-enhancing), were presented to each participant, who answered questions on pro-environmental intentions for one self-enhancing and one self-transcendent value frame (presented in random order), of which one randomly referred to conserve energy and the other to use public transit (serving as our main dependent variables). No differences emerged in the two types of behaviors, thus we merged the scores. Finally, participants were debriefed and dismissed.

##### Data analysis

We used a mixed-model ANOVA and subsequent *t*-test to test the main hypotheses in Experiment 1; the decision of using such statistical analysis techniques is justified by the experimental nature of our procedures, were the random grouping of participants was necessary.

#### Results

**FIGURE 1:**
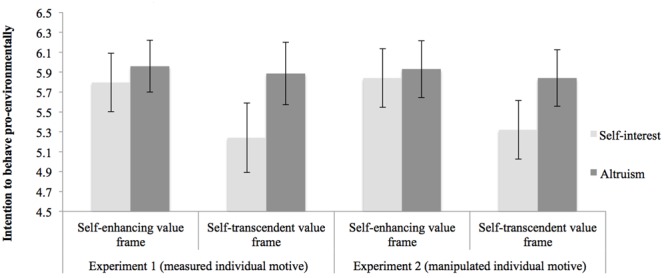
Results of Experiments 1 and 2. Individual attitude (self-interest vs. altruism) by situational value frame (self-enhancing vs. self-transcendent) interaction effect on intention to behave pro-environmentally. In Experiment 1, individual attitude has been measured; in Experiment 2, individual attitude has been manipulated. The dependent variable is measured from 1 to 7. Error bars represent 95% CI of the mean.

The first survey measured basic socio-demographic variables and dispositional attitudes toward the environment (Schultz et al., 2005) (self-interest vs. altruism). Then, to test for our main hypothesis, the second survey presented two scenarios in which we manipulated the situational value frame (self-enhancing vs. self-transcendent) and relevant pro-environmental behavior. Each participant answered questions about pro-environmental intentions in the self-enhancing and in the self-transcendent value frame (presented in random order), of which one randomly referred to conserving energy and the other to using public transit. The main dependent variable here was the intention to conserve energy and use public transit (as described in the method section). A mixed model ANOVA tested the difference in intentions to act pro-environmentally between dispositional environmental concern (self-interest vs. altruism; between-subjects variable) and the experimental effect of the situational value frame (self-enhancing vs. self-transcendent; within-subjects condition). Confirming H1, results showed a significant interaction effect between the two variables, *F*(1,120) = 3.80, *p* = 0.05, η^2^ = 0.03. **Figure 1** (Experiment 1) shows the subsequent two pairwise comparisons. Specifically, a first comparison showed that for self-interested individuals, when the behavior was presented in a self-enhancing value frame (i.e., when the message highlighted a personal gain), participants reported more pro-environmental intentions (*M* = 5.80, *SD* = 1.18, *N* = 54) than when the situational value frame was self-transcendent [*M* = 5.24, *SD* = 1.52, *N* = 54, *t*(120) = 3.00, *p* < 0.001, *d* = 0.58], confirming our hypothesis. Among individuals classified as more strongly altruistic, no significant differences emerged in pro-environmental intentions for the two different value frames [*t*(120) = 0.45, *p* > 0.05; self-enhancing: *M* = 5.96, *SD* = 1.03, *N* = 68; self-transcendent: *M* = 5.89, *SD* = 1.12, *N* = 68]. Importantly, to further investigate the effect, a second pairwise comparison showed that when the situational value frame was self-enhancing, no substantial difference emerged in the intention to behave pro-environmentally between individuals with an altruistic environmental disposition (*M* = 5.96, *SD* = 1.03, *N* = 68) or a self-interested disposition [*M* = 5.80, *SD* = 1.18, *N* = 54, *t*(120) = 0.63, *p* > 0.05]. On the contrary, consistent with our hypothesis, when the situational message frame was self-transcendent, altruistically oriented individuals intended to behave more pro-environmentally (*M* = 5.89, *SD* = 1.12, *N* = 68) than did those oriented toward self-interest [*M* = 5.24, *SD* = 1.52, *N* = 54, *t*(120) = 2.49, *p* < 0.001, *d* = 0.45].

Taken together, these results show that a relationship between the self-enhancement and self-transcendence values and individuals’ willingness to engage in pro-environmental behaviors (Schultz et al., 2005) is not a relation based on a simple correspondence between these two variables. Our results from the first experiment suggest that the structure of environmental concerns could be inclusive and hierarchical, also according with a more general environmental psychology’s systemic perspective of interdependence between individuals and the environment (Lewin, 1951; Bronfenbrenner, 1977; Bonnes and Bonaiuto, 2002; Winkel et al., 2009). In practice, appealing to self-interest would increase the likelihood that both self-interested and altruistic individuals would engage in green behaviors, contrary to the simple correspondence explanation (or “fit explanation”) between values and behaviors.

### Experiment 2

#### Aims and Hypothesis

The general aim of Experiment 2 was to further explore the hierarchical structure of environmental concerns, and to test the possibility of manipulating environmental concerns and to replicate the results emerged in Experiment 1. While Experiment 1 tested the effect of situational motive and environmental dispositional concern on the intention to enact pro-environmentally measuring one’s environmental concern as an individual dispositional characteristic, Experiment 2 tested the effect of situational motive and environmental concern on the intention to enact pro-environmentally within a full experimental methodology, manipulating both the situational value frame *and* environmental concerns. We hypothesized that individuals who were experimentally manipulated with materials designed to activate self-interested environmental concern would show a greater egoistic concern compared to those exposed to the altruism condition, who would show greater biospheric concern (H2a). This is a basic replication of previously reported findings. However, we also hypothesized that individuals in the self-interest experimental condition would be more willing to behave green when a message frame highlighted the personal benefits of the action (self-enhancing value frame); conversely, for individuals in an experimental condition that induced altruism, participants would be equally likely to engage in behaviors that were framed with personal benefits (self-enhancing value frame) or environmental benefits (self-transcendent value frame; H2b). In order to test this hypothesis, the pattern of results that emerged from Experiment 1 should be replicated, but this time using an experimental manipulation of environmental concern. Experiment 2 will therefore provide evidence that environmental concerns can be manipulated, and that they could also be defined as specific and situationally activated tendencies or attitudes toward the environment rather than being a stable dispositional factor only. More generally, Experiment 2 will provide further evidence supporting the idea that the structure of environmental concern is inclusive and hierarchically organized, and that this structure may drive behaviors in different ways when a specific value frame or motive is contextually activated.

#### Methods and Materials

##### Participants

A total sample of 156 (female: 94.2%; average age: 20.3 years; ethnicity: 39.4% White, 42.9% Latino, 4.5% Black, 7.7% Asian, 5.1% Other) undergraduate students from CSUSM were recruited during the spring semester of 2014.

##### Experimental procedures

In Experiment 2, the procedure was similar to Experiment 1, except that we added the environmental concern manipulation before the survey administration, according to a comparable procedure developed previously (Sevillano et al., 2007). Specifically, participants were randomly assigned to view one of two kinds of images, either people in nature (self-interest condition) or animals being harmed by the consequences of human activity (altruism condition). Instructions asked participants to look at four pictures (1 min each), to take the perspective of the subjects shown in the image (Sevillano et al., 2007), and then to respond some related questions (manipulation check on perspective taking). The stimuli were composed by four color pictures similar to those used in previous studies (Schultz, 2000): each picture represented either harmed animals/endangered nature or persons enjoying nature, respectively, for the manipulation of altruism and self-interest. Each picture was followed by a two-line description of the subject(s) and the scene represented. The pictures in the harmed animals condition represented a wild elephant being caught, oil from the Gulf spill covering the beach and local birds, a tree being cut in the rainforest, and a seal caught in a fishing net; in the people in nature condition, pictures represented girls surfing at the beach, a snowboarder watching the landscape, a group of people rafting, and a couple camping in the forest. The images (about 11 cm × 17 cm each) were printed in color in HD and presented in transparent plastic folders. To check the manipulation and similarly to Experiment 1, participants responded to the environmental concern scale (Schultz et al., 2005) (serving as main dependent variable in this phase); finally, to test for within-subjects effect, the experimental procedure used in Experiment 1 was replicated: two scenarios were presented to each participant where we manipulated the situational value frames. For each of the two value frames, two different pro-environmental behaviors (conserve energy and use public transit) were also manipulated and presented randomly. The resulting four sets of combinations were randomly assigned to participants.

##### Data analysis

In Experiment 2, a one-way ANOVA first, and then a mixed-model ANOVA and subsequent *t*-test were used to test for the effectiveness of our manipulation and for our main hypotheses; again, such statistical analysis techniques were used because of the experimental nature of our procedures.

#### Results

Previous results from Experiment 1 showed that the situational message frame of the behavior interacted with a person’s type of environmental concern in determining pro-environmental behavior. However, this experiment did not answer the question of whether or not these concerns could be situationally defined. In order to answer this question, in Experiment 2 we added a phase where we manipulated environmental concerns (Sevillano et al., 2007) before replicating the procedure of Experiment 1. Here, our main dependent variables were the environmental concerns first, and then the intention to conserve energy and use public transit (the same used in Experiment 1). Confirming H2a, results showed that participants who were shown pictures of individuals enjoying the nature (self-interest condition) subsequently showed higher levels of egoistic environmental concern compared to those shown pictures of animals being harmed in nature (altruism condition), who reported greater levels of biospheric environmental concern, *F*(1,153) = 8.57, *p* = 0.004. Moreover, the pattern of results emerged to test H2b was consistent with what emerged in Experiment 1 (**Figure 1**, Experiment 2): when the situational message frame was self-transcendent, altruistic individuals intended to behave more pro-environmentally (*M* = 5.84, *SD* = 1.29, *N* = 81) than self-interested ones [*M* = 5.32, *SD* = 1.28, *N* = 74, *t*(153) = 2.32, *p* < 0.02, *d* = 0.38]. However, when the situational value frame was self-enhancing, no substantial difference emerged in the intention to behave pro-environmentally between altruistic individuals (*M* = 5.93, *SD* = 1.29, *N* = 81) and self-interested ones [*M* = 5.84, *SD* = 1.29, *N* = 74, *t*(153) = 0.40, *p* > 0.05]. Again, the results, this time derived from the analysis of manipulated environmental concerns, support our model of an inclusive structure of environmental concerns.

Presenting a situation framed in a self-transcending value elicited greater green behaviors in altruistic individuals (suggesting a higher level of inclusion for altruism); while presenting a self-enhancing framed situation elicited green behaviors both in self-interested and altruistic individuals (suggesting a lower level of inclusion for self-interest). Moreover, the fact that it was possible to manipulate environmental concerns opens new possibilities in studying this social psychological variable as a situation-dependent factor or motive (Schultz, 2001) that could be primed or activated, rather than as a more stable individual disposition only.

### Experiment 3

#### Aims and Hypothesis

The general aim of Experiment 3 was to replicate findings of the previous studies within an experimental setting that uses an actual pro-environmental behavior as the main dependent variable, rather than behavioral intentions. While Experiments 1 and 2 tested the effect of environmental concerns (respectively, dispositional and manipulated) and situational motives on intentions to enact pro-environmentally, Experiment 3 will test the effect of situational motives and environmental concerns on a real behavior, that is signing up to participate in a beach clean up event. According to our previous results, we hypothesized that people holding an egoistic environmental concern would behave green when a self-enhancing message frame matched their self-interested concern, while both self-enhancing and self-transcendent message frames would increase green behaviors of individuals with more altruistic concerns. However, also according to the results in the two previous studies, we expected that a significant reduction in green behavior would emerge in the self-transcendent value frame condition for individuals with self-interested environmental concerns. Therefore, we specifically hypothesized that (H3): self-interested individuals would decrease their behavior in the self-transcendent value frame (vs. self-enhancing), while no differences would emerge among altruists. Furthermore, while we did not expect any difference to emerge in the self-enhancing condition, in the self-transcendent value frame condition, we predicted that individuals with more altruistic environmental concerns would behave more pro-environmentally than individuals with egoistic concerns.

#### Methods and Materials

##### Participants

Based on a power analysis (Cohen, 1992), we aimed at reaching a sample of 180 subjects. However, 161 undergraduate students of the California State University, San Marcos (CSUSM) were recruited during the end of spring semester 2014 (May). Of those who participated in the experiment, six were excluded from the final dataset due to previous participation in the research or misconduct in the experiment. A final sample of *N* = 155 (female: 70%; average age: 19.8 years; ethnicity: 42.6% White, 39.4% Latino, 4.5% Black, 8.4% Asian, 3.9% Other) was therefore used for the data analysis.

##### Measures

A paper-and-pencil survey measured first three socio-demographic variables: gender, age and ethnicity. Then, the environmental concerns scale (Schultz, 2001; Schultz et al., 2005) consisted of 12 items with a 7-point response Likert scale, measuring environmental concerns (egoistic, social, and biospheric), with four items measuring each concern. The three subscales showed good reliability, all α > 0.85. After this first section, one of the two brochures was presented. For each brochure, participants decided whether to fill out an application form, providing name, email, date, and answering three items measuring engagement in participation (how important are beach clean up events such as this one? how interested would you be in participating in a beach clean up day like this one? would you be willing to participate in a beach clean up day like this one?). At the bottom of the form, they were asked to sign up and participate in the beach clean up by checking one option for their participation (one item measuring participation: yes, maybe, no) and signing the application form.

##### Experimental procedures

In Experiment 3, we tested a 2 (situational self-enhancing vs. self-transcendent motive) × 2 (self-interest vs. altruism) full factorial experimental design. Our participants first filled in the survey, then were invited to participate in an off campus beach clean up event which was unrelated to their university duties, and finally responded some general questions about the beach clean up. As previously reported, the first survey measured basic socio-demographic variables, environmental concerns (Schultz et al., 2005) as the operationalization of self-interest and altruism, and some other unrelated social-psychological variables. Then, in order to manipulate for the situational value frame, we presented participants an informative brochure about a beach clean up event organized by a local non-profit organization, which would have been held in the North San Diego County, CA, United States (about 18 miles away from campus). Participants were therefore informed about the event, which was presented as an extra activity neither related to their university duties nor to the experiment, and they were invited to participate. They were free to accept or decline without any direct consequence for them, except for those made explicit in the brochures. The two brochures (of which one was randomly presented to each participant), created from real material used in previous local beach clean up communication campaigns, were framed as a self-enhancing vs. self-transcendent event stating a call to action: the participation in a local beach clean up. The self-enhancing brochure stated “50$ gift card! Free lunch! Lot’s of fun!” and “do it for yourself” and had pictures of individuals having fun at the beach (with features similar to the “people in nature” pictures used in Experiment 2), eliciting self-enhancing motives; the self-transcendent brochure had pictures of harmed animals and stated that “1,102,042 bottle caps found since 1985” and “it’s about nature,” eliciting self-transcendent motives. By this procedure, the specific self-transcendent vs. self-enhancing value was therefore elicited in each participant [a pilot-tested manipulation check showed that our manipulation was effective in manipulating the situational value frame, *t*(28) = 2.43, *p* = 0.02], who decided to sign up or not for the participation by checking one of the presented options for their participation (yes, maybe, not) and eventually signing the application form. After their decision, only participants who selected “yes” or “maybe” and signed the brochure received an official invitation card (comparable to the brochures) to the beach clean up, signed both from the researcher and the participant, who finally answered few questions about the event. The decision to accept (by signing up) or decline the invitation served as our main dependent variable. Then, participants were debriefed and probed for suspicion.

##### Data analysis

In order to test our main hypotheses, we used a logistic regression and subsequent slope analysis to probe for the expected specific effect.

#### Results

**FIGURE 2:**
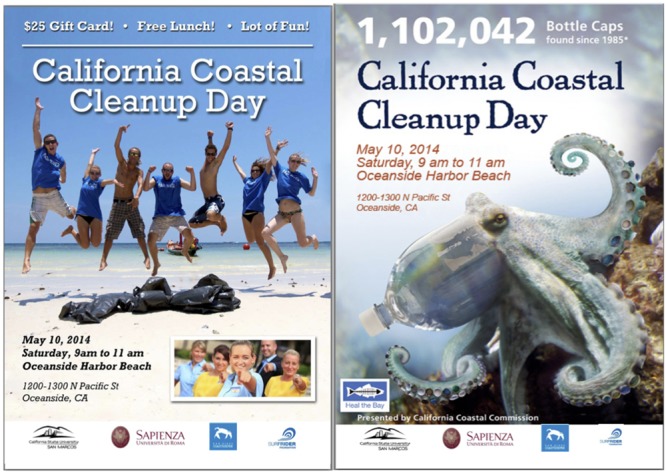
Self-enhancing and self-transcendent value frames used in Experiment 3.

**FIGURE 3:**
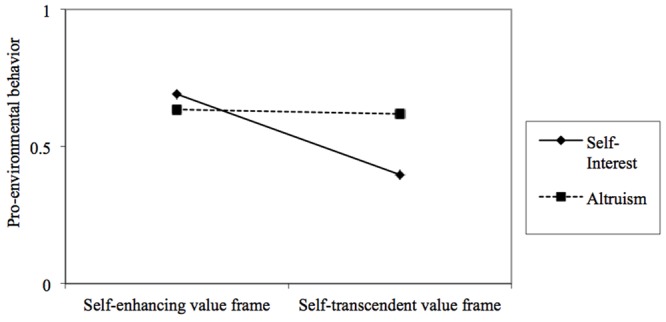
Results of Experiment 3. Individual attitude (self-interest vs. altruism) by situational value frame (self-enhancing vs. self-transcendent) interaction effect on pro-environmental behavior. The slope analysis shows significantly different slopes at the 95% CI.

In Experiment 3, after completing a survey where we measured environmental concerns and being randomly assigned to one of two experimental conditions (self-enhancing vs. self-transcendent), participants were invited to join in a beach clean up day (**Figure 2**), organized by a local non-profit organization. In line with the previous two studies, we framed the invitation with either a self-enhancing or a self-transcendent motivation: a manipulation check showed that our participants perceived the event presented in a self-transcendent value frame (vs. self-enhancing) to be more beneficial for the environment (*M* = 2.10; *SD* = 1.64; *N* = 76) than for the person herself [*M* = 1.61; *SD* = 1.38; *N* = 76), *t*(150) = -2.04, *p* = 0.04]. Then, to test for our main hypothesis, we measured sign up rates for the beach clean up, using this behavior as our main binary dependent variable. We expected a significant reduction of the behavior among self-interested individuals in the self-transcendent condition (vs. self-enhancing). Furthermore, we predicted that no difference would emerge in the self-enhancing condition between self-interested and altruistic individuals, but that lower levels of behavior would emerge for self-interested individuals (compared to altruistic ones) in the self-transcendent condition. To test our hypotheses, we ran a logistic regression analysis using PROCESS (Hayes, 2013): sign up rate was predicted by situational value frame (either self-enhancing or self-transcendent; situational frame was dummy-coded in the analysis with self-enhancing = 0 and self-transcendent = 1), environmental concern (with continuous scores increasing from self-interest to altruism), and their interaction. Results showed that the tested moderation model (model 1; see Hayes, 2013) significantly predicted sign up rates, χ^2^ (3) = 8.43, *p* = 0.038, Nagelkerke *R*^2^ = 0.07. Also, for completeness, we computed direct main effect of each predictor on the outcome variable using a hierarchical logistic regression analysis (Brambor et al., 2006). Specifically, results showed a significant conditional effect of manipulated situational value frame on signing up, *b* = -0.65, *p* = 0.049, such that participants would sign up for the beach clean up event significantly more in the self-enhancing (vs. self-transcendent) condition (direct effect: *b* = 0.65; *p* = 0.04); while no significant conditional effect emerged for environmental concern, *b* = 0.12, *p* = 0.34 (direct effect: *b* = 0.15; *p* = 0.23). Importantly, a marginally significant interaction effect was found, *b* = 0.44, *p* = 0.09. The subsequent slope analysis probed the interaction and the test of conditional effects confirmed our H3 (**Figure 3**): for individuals holding an altruistic environmental concern (operationalized as the mean-centered environmental concern score +1 SD), a change in the situational value frame did not affect their behavior, *b* = -0.07, *p* = 0.88. However, for individuals holding a self-interested environmental concern (operationalized as the mean-centered environmental concern score -1 SD), a change in the situational value frame (from self-enhancing to self-transcendent) significantly reduced their behavior, *b* = -1.22, *p* = 0.01 (95% CI = -2.17; -0.27).

**FIGURE 4:**
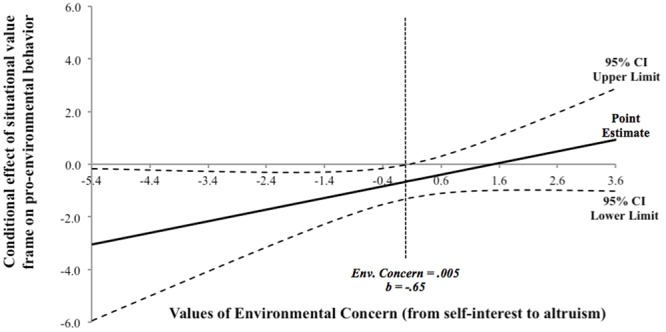
Johnson–Neyman estimation of conditional effect of situational value frame on pro-environmental behavior at specific values of environmental concern (Experiment 3). For environmental concern more oriented toward self-interest (i.e., environmental concern <0.005), the change from self-enhancing to self-transcendent situational value frame significantly decreases the target pro-environmental behavior. The point estimation is significant at the 95% CI.

Taken together, the results showed that while no differences emerged among altruists, a significant change occurred in self-interested individuals according to the different situational value frames. Specifically, a reduction in their behavior occurred in the self-transcendent condition. To further understand this effect, in addition to the logistic regression model and slope analysis, we used the Johnson–Neyman technique (Hayes, 2013; Johnson and Fay, 1950; Neyman, 1936) to ascertain where on the environmental concern continuum change (from self-interest to altruism) the effect of situational value frame transitions from statistically significant to not significant. Results showed that the effect of the situational value frame on sign up rate was significant for participants with environmental concern scores below 0.005 (**Figure 4**). Of participants who were more oriented toward self-interest, 47.82% were significantly more motivated to enact pro-environmental behaviors in the self-enhancing situational value frame than in the self-transcendent one. In other words, both individuals oriented toward self-interest and those oriented toward altruism engaged in the green behavior when the situation was self-enhancing, while altruistic individuals were more likely to engage in the behavior in the self-transcending situation.

To further explore the psychological pathways for pro-environmental behavior, a series of *post hoc* analyses were conducted. First, a regression analysis [*F*(3,102) = 3.41, *p* = 0.02, *R*^2^ = 0.09] showed that individuals held a better overall evaluation of the event (they found it more important, interesting and appealing; α = 0.93) according to their altruistic environmental concern (direct effect: *b* = 0.27, *p* = 0.01), but irrespective of the self-enhancing or self-transcendent value frame (direct effect: *b* = 0.37, *p* = 0.16; interaction effect: *b* = -0.27, *p* = 0.21). A second regression analysis [*F*(3,155) = 2.64, *p* = 0.05, *R*^2^ = 0.05] showed that individuals found information about the event to be more personally motivating in the self-enhancing frame (direct effect: *b* = -0.60, *p* = 0.03), but irrespective of their self-interested or altruistic environmental concern (direct effect: *b* = 0.17, *p* = 0.11; interaction effect: *b* = 0.10, *p* = 0.65). These unplanned analyses showed interesting results. Although individuals seemed to favor the event according to their dispositional altruistic environmental concern, their motivation to participate was instead positively related to the self-enhancing situational value frame. Therefore, while individuals might embrace a given dispositional environmental concern and being, generally speaking, more oriented either toward self-interest or altruism, a situational value frame focused on the enhancement (rather than on the transcendence) of the self might be more motivating for a greater audience. These results, although not hypothesized, further support our IMEC model: on the whole, altruists perceived a pro-environmental event (such as a beach clean up day) to be more important, interesting and appealing than did self-interested participants. However, the event was personally motivating for both altruists and self-interested individuals when presented in a self-enhancing situational value frame. In other words, while a typical self-transcendent event was more appealing for altruistic individuals (suggesting a higher level of inclusion for altruism), presenting such an event in a situational self-enhancing value frame was motivating both for altruistic and self-interested individuals (suggesting a lower level of inclusion for self-interest).

## Discussion

### Promoting Collective Environmental Action

The tension between altruistic and self-interested foundations for pro-environmental behavior has been a longstanding point of discussion. On the one hand, previous research has shown that an altruistic orientation is associated with heightened levels of environmental concern and with pro-environmental behavior, whereas a egoistic self-interested orientation tends to be negatively associated with environmental concern and action (Schultz et al., 2005; Gifford, 2011, 2013; Steg and de Groot, 2012; Gifford and Nilsson, 2014). In addition, research has shown that appealing to monetary incentives vs. environmental ones is not a particularly effective strategy for promoting green behaviors (Bolderdijk et al., 2013; Asensio and Delmas, 2015). But on the other hand, some researchers have argued that appealing to the societal and economic (i.e., self-interested) benefits could be more effective than focusing on ecologic outcomes or environmental (i.e., altruistic) issues, for example because they could enhance one’s own status (Griskevicius et al., 2010) or motivate behavioral change among climate change deniers (Bain et al., 2012). Because the psychological structure of values is universal (Schwartz and Bilsky, 1987, 1990; Schwartz, 1992), a strong theoretical model explaining how different value-based frames could motivate broad-based pro-environmental behaviors could help to inform conservation campaigns. This, in turn, will shed light on how to promote widespread collective environmental action. In this paper, we present evidence that both self-interest *and* altruism can provide pathways to pro-environmental behavior (Delmas et al., 2013), introducing that this is due to different yet inter-related psychological bases. Our studies were guided by the Inclusion Model of Environmental Concern, which states that environmental concerns are organized in a systemic and hierarchical way: egoistic concerns (lower level of inclusion), which represent the operationalization of what is commonly called self-interest, are embedded within biospheric concerns (higher level of inclusion), or the operationalization of altruism. Our data are compatible with the model, and not with a simpler correspondence explanation. More specifically, individuals oriented toward self-interest are likely to engage in pro-environmental behaviors in a self-enhancing situational value frame rather than in a self-transcendent one—suggesting a lower level of inclusion; whereas altruistic oriented individuals are likely engage in pro-environmental behaviors both in a self-enhancing and self-transcendent situational value frame—suggesting a higher level of inclusion. This is also in line with the notion that pro-social behavior could be motivated by inner self-oriented concerns, such as social reputation or self-respect (Bénabou and Tirole, 2006). More generally, our results are in line with the broader idea that human behavior and motivation depend on the hierarchical evolution, structure, and functioning of the human brain (Edelman, 1987; MacLean, 1990), from the inner part (i.e., the reptilian brain or basal ganglia) oriented toward the most instinctive and self-oriented behaviors, to the external part (i.e., the neocortex) oriented toward the culturally shaped, metacognitive and self-transcendent behaviors.

Although the reported results do show a pathway to pro-environmental behavior through self-interest, it is important to be cautious in deriving policy implications from them. First, in the self-enhancing condition, self-interest oriented individuals increased their green action on average by 15.7%. Second, following the strength of inference approach (Frank et al., 2013), our results show an 88% likelihood to be generalized to other contexts of application. However, we do not advocate for the extensive promotion of self-enhancing values and/or self-interest for behavioral change to protect the environment (Steg and Vlek, 2009; Bolderdijk et al., 2013) to the detriment of self-transcendence and altruism. Rather, we suggest that both self-enhancing *and* self-transcendent based intervention programs could be beneficial in promoting collective environmental action.

Moreover, even though individual actions are often driven by evolutionarily adaptive psychological biases related to self-enhancing goals (van Vugt et al., 2014), self-enhancing frames based on extrinsic motivation and rewards, such as financial incentives, may in fact have negative longer-term consequences (Vohs et al., 2006). Thus, in the process of planning policies and interventions, the balance between intrinsic/extrinsic motivation and self-enhancement/self-transcendent values promotion must be taken in to account:

• When money is made salient individuals tend to behave more selfishly (Vohs et al., 2006), no matter what the goal of their actions.
• Appealing to economic self-interest is unlikely to promote an ecological self-concept (Bolderdijk et al., 2013), and research has shown that individuals strive for consistency between their behavior and this self-concept (Aronson, 1992).
• Individuals may behave pro-environmentally for non-environmental reasons, such us gaining social status (Griskevicius et al., 2010) or being healthy (Gifford, 2011, 2013), and many times individuals behave pro-environmentally even without knowing they are doing so (Gifford, 2013).
• Leveraging self-enhancing motives may, in the long run, undermine the intrinsic motivation to behave pro-environmentally (Kollmuss and Agyeman, 2002; Schwartz et al., 2015), resulting in lower overall levels of pro-environmental behavior.
• Not every green behavior can be promoted via self-interest (Stern and Dietz, 1994).
• Pro-environmental behavior that is motivated through self-interest is unlikely to spillover into other, related behaviors (Thøgersen and Crompton, 2009; Bolderdijk et al., 2013; Evans et al., 2013; Steinhorst et al., 2015).

Finally, although our results showed that both altruists *and* self-interested individuals can be motivated to engage in pro-environmental behaviors, it is not clear whether or not a target pro-environmental behavior enacted in response to different situational value frames implies the possibility of attitudes change (Crano and Prislin, 2008)—or, possibly, values change in the long run. Our data show that a self-enhancing situational value frame can increase pro-environmental behavior among self-interested individuals, who would otherwise have lower levels of pro-environmental behavior. Yet, it seems that message framing (being either self-enhancing or self-transcendent) exerts a small or no impact on altruists, who would perform the pro-environmental behavior anyway. According to the IMEC, because self-interest is embedded within altruism, we speculate that: (a) altruistic individuals can in fact be motivated by self-interested reasons, but this does not necessarily imply attitude change toward self-interest; (b) self-interested individuals can be motivated to enact green behaviors in a self-enhancing value frame, yet this does not imply attitude stagnation. Further research should therefore investigate at least two main aspects: (1) the relationship between self-interested and altruistic environmental concerns or attitudes, engagement in pro-environmental behaviors and attitude change; (2) within the IMEC paradigm, the relationship between self-interested and altruistic environmental concerns or attitudes and the perception of different situational self-enhancing and self-transcendent value frames.

### Practical Implications and Conclusion

Given the unintended side-effects summarized above, communication campaigns should not be exclusively focused on financial and extrinsic motives in order to promote pro-environmental behaviors (Bolderdijk et al., 2013; Evans et al., 2013). In order to promote durable long-term changes in behavior, conservation programs that use intrinsic motivators are likely to be more effective (Deci and Ryan, 1985; Ryan and Deci, 2000a,b; Griskevicius et al., 2010; Boedecker et al., 2013; Schwartz et al., 2015). However, it could be possible that in certain circumstances—for example, if cognitive dissonance arises (Festinger, 1957) or if a new ambivalent attitude emerges (Petty et al., 2006; Hohman et al., 2014)—the engagement in pro-social behaviors might positively shape related attitudes (Crano and Prislin, 2008; Bohner and Dickel, 2011). Within this reciprocal process, and in line with the theory of the commons and to the general literature on social dilemmas (Dietz, 2005), focusing on specific contexts where self-interest can be invoked in the service of the environment can play an important role in promoting collective environmental action. Highlighting self-enhancing reasons first, in order to motivate a greater audience to engage in pro-environmental behaviors, and then moving to a more self-transcendent value-based communication (Asensio and Delmas, 2015; Steinhorst et al., 2015; Steinhorst and Matthies, 2016), could be an effective wider and more complex process to shape individuals’ perspectives or values (Bardi and Schwartz, 2003) and to spur global behavioral change toward a more sustainable future encompassing a much broader audience across different systems of varied values and concerns.

## Ethics Statement

This study was carried out in accordance with the recommendations of the Institutional Review Board for the Protection of Human Subjects (IRB) of the California State University San Marcos with written informed consent from all subjects. All subjects gave written informed consent in accordance with the Declaration of Helsinki. The protocol was approved by Dr. Susan Thompson, IRB Chair.

## Author Contributions

SDD, PS, and MB jointly designed the research question; SDD collected data and prepared data analysis to discuss with PS; SDD drafted the manuscript; PS and MB provided feedback on the manuscript.

## Conflict of Interest Statement

The authors declare that the research was conducted in the absence of any commercial or financial relationships that could be construed as a potential conflict of interest.
