# Supplementary material for: Protecting the Environment for Self-interested Reasons: Altruism Is Not the Only Pathway to Sustainability
Source: Front Psychol. 2017 Jun 28;8:1065. doi: 10.3389/fpsyg.2017.01065 (PMC5487490; doi:10.3389/fpsyg.2017.01065)
Supplement: Supplementary file 1 [file Data_Sheet_1.docx]

*Frontiers in Psychology*

Supporting Information for

**Protecting the Environment for Self-interested Reasons:**

**Altruism is not the only Pathway to Sustainability**

Stefano De Dominicis^1-2^, P. Wesley Schultz^3^, Marino Bonaiuto^1-4^

^1^ Department of Business and Management, Università LUISS Guido Carli, Viale Romania 32, 00197, Roma, Italy.

^2^ Dipartimento di Psicologia dei Processi di Sviluppo e Socializzazione, Sapienza Università di Roma, Via dei Marsi, 78, 00185, Roma, Italy.

^3^ Department of Psychology, California State University San Marcos, 333 S. Twin Oaks Valley Rd., San Marcos, CA 92096-0001.

^4^ CIRPA - Centro Interuniversitario di Ricerca in Psicologia Ambientale, Sapienza Università di Roma, Via dei Marsi, 78, 00185, Roma, Italy.

**Contents of this file**

Text S1: Scenario value frame manipulation in Experiment 1 and Experiment 2

**Introduction**

The Text S1 provides the 4 different scenarios used for the manipulation of main independent variables (namely, value frame and pro-environmental behavior) used in Experiment 1 and Experiment 2. The combination of the two independent variables is:

- Scenario A1 (self-enhancement X energy conservation);
- Scenario B1 (self-transcendence X energy conservation);
- Scenario A2 (self-enhancement X public transportation);
- Scenario B2 (self-transcendence X public transportation);

Text S1. Scenario value frame manipulation in Study 1 and Study 2

*Scenario A1 (self-enhancement X energy conservation):*

Imagine you are a first year college student. You live in an on-campus student housing unit. You have your own private room, with a small kitchenette, and a private bathroom. The student housing office charges you with costs for the rent and electricity consumption each month. After the first semester, the school introduced a new program to reduce energy consumption, and this program is tailored specifically for the reduction of the electricity consumption in each university building. For on-campus student housing, the goal is to reduce the energy consumption by 25%. In order to achieve this goal, the housing office promotes energy conservation incentives. Incentives allow students to earn a considerable amount of reduction in their electricity bill: if you conserve 25% of your average for the month, the school will match that savings and lower the cost of your bill as if you had saved half of your average. Each month you conserve 25% of your past average energy consumption, you will get a 50% reduction of your bill. Let’s say in the first semester you paid on average $40.00 on your energy bill per month. With this incentive, each month that you will conserve energy for a total amount of $10.00 worth of energy on your bill, you will get an extra $10.00, paying only $20.00 per month instead of $40.00. This situation would be very beneficial to you.

*Scenario B1 (self-transcendence X energy conservation):*

Imagine you are a first year college student. You live in an on-campus student housing unit. You have your own private room, with a small kitchenette, and a private bathroom. The student housing office charges you with costs for the rent and electricity consumption each month. After the first semester, the school introduced a new program to reduce energy consumption, and this program is tailored specifically for the reduction of the electricity consumption in each university building. For on-campus student housing, the goal is to reduce the energy consumption by 25%. In order to achieve this goal, the housing office promotes energy conservation feedback on carbon equivalencies so students will have a specific knowledge of their impact on the environment: if you conserve 25% of your average for the month, you will stop producing a considerable amount of greenhouse gas emissions equivalent to 1.6 gallons of gasoline and an amount of carbon sequestered from the atmosphere equivalent to 500 square feet of U.S. forest per year. With this conservation, for six months you will save a total amount of 9.6 gallons of gasoline in the atmosphere with an effect equal to 3000 square feet of forest in one year. This situation would be very beneficial for the environment.

*Scenario A2 (self-enhancement X public transportation):*

Imagine you live in a big city. You live in a nice and central neighborhood, you have to commute everyday to work. After your first year in this city, the municipality introduces a new public transportation program, to reduce carbon emissions and improve air quality. In order to move toward a more efficient mobility plan in the city, the municipality promotes the use of public transportation with incentives. Incentives allow residents to have a convenient monthly pass to use public transportation. You discover that the monthly public transit pass is 75% less expensive than using your car and that a new bus line will directly connect your home and your workplace. With this new bus line, you will spend about the same amount of time to get to your job compared to the time you use to spend to get there when using your car. Thus, for each month using public transportation you will save up to 75% on your mobility expenses, you will spend about the same amount of time for your commute, but you will not have no stress nor risks related to driving in traffic. This situation would be very beneficial to you.

*Scenario B2 (self-transcendence X public transportation):*

Imagine you live in a big city. You live in a nice and central neighborhood, you have to commute everyday to work. After your first year in this city, the municipality introduces a new public transportation program, to reduce carbon emissions and improve air quality. In order to move toward a more efficient mobility plan in the city, the municipality promotes the use of public transportation with feedbacks on carbon equivalencies. A city that uses public transportation has a strong positive impact on the environment, making the city a better place to live in, with a cleaner air and less pollution. You discover that a new bus line will directly connect your home and your workplace. With this new bus line, you will reduce your emissions in the atmosphere by 75% compared to the emissions you produce with your car. For each month of public transportation use, you will significantly reduce the greenhouse gas emissions in the environment (up to 75%), making your city a better place to live in and also protecting the environment: you will save an amount of greenhouse gasses in the atmosphere equivalent to 300 metric tons, which is about 35 gallons of gasoline in the atmosphere and an amount of carbon sequestered from the atmosphere, equivalent to about 18000 acres of forest in one year. This situation would be very beneficial for the environment.
